# Synergistic use of satellite, legacy, and *in situ* data to predict spatio-temporal patterns of the invasive *Lantana camara* in a savannah ecosystem

**DOI:** 10.3389/fpls.2025.1593110

**Published:** 2025-08-11

**Authors:** Lilly Theresa Schell, Emma Evers, Sarah Schönbrodt-Stitt, Konstantin Müller, Maximilian Merzdorf, Drew Arthur Bantlin, Insa Otte

**Affiliations:** ^1^ Department of Remote Sensing, Institute of Geography and Geology, University of Würzburg, Würzburg, Germany; ^2^ Conservation and Research Department, Akagera National Park, Kayonza, Eastern Province, Rwanda

**Keywords:** *Lantana camara*, species distribution, random forest, invasive species, google earth engine

## Abstract

Modeling species distributions is critical for managing invasive alien species, as reliable information on habitat suitability is essential for effective conservation and rehabilitation strategies. In this study, we modeled the suitable habitat and potential distribution of the notorious invader *Lantana camara* in the Akagera National Park (1,122 km²), a savannah ecosystem in Rwanda. Spatiotemporal patterns of *Lantana camara* from 2015 to 2023 were predicted at a 30-m spatial resolution using a presence-only species distribution model, implementing a Random Forest classification algorithm and set up in the Google Earth Engine. The model incorporated Sentinel-1 SAR, Sentinel-2 multispectral data, anthropogenic predictors, and *in situ* presence data of *Lantana camara*. A maximum of 33% of the study area was predicted as a suitable *Lantana camara* habitat in 2023, with higher vulnerability in the central, northern, and southern Akagera National Park. The change detection analysis revealed an increase in habitat suitability in the northeastern sector and a decrease in the southwestern part of the park over the study period. The model's predictive performance was robust, with AUC_ROC_ values ranging from 0.93 to 0.98 and AUC_PR_ values ranging from 0.79 to 0.94. Key factors influencing *Lantana camara* habitat suitability in the study area are the road network, the elevation, and soil nitrogen levels. Additionally, the red edge, shortwave, and near-infrared spectral bands were identified as essential predictors, highlighting the efficacy of combining remote sensing and anthropogenic data with machine learning techniques to predict invasive species distributions. These findings provide valuable guidance for developing effective conservation strategies to protect savannah ecosystems and mitigate the spread of *Lantana camara* in the future.

## 1 Introduction

Invasive alien species (IAS) pose a significant and accelerating threat to global biodiversity and local ecosystems (Raghubanshi et al., 2005; Kandwal et al., 2009). Although invasion processes are a natural phenomenon, global warming, globalization, and the resulting rapid changes in the natural habitats of plants and animals, such as fragmentation, have accelerated and intensified the rate of invasions in the biosphere over the last century (Raghubanshi et al., 2005; Meyerson and Mooney, 2007; Mondal et al., 2021). Today, invasion processes represent the second-largest threat to global biodiversity after the destruction of ecosystems and habitats (Dueñas et al., 2018). The ecological consequences are becoming increasingly evident and require urgent measures to regulate and conserve natural ecosystems (Raghubanshi et al., 2005; Kandwal et al., 2009; Ruwanza and Shackleton, 2016).

*Lantana camara* L. (sensu lato) is an invasive weed in the *Lantana* genus of the Verbenaceae family with high phenotypic plasticity and genetic diversity (Goyal and Sharma, 2015; Gunasekara and Ranwala, 2018). Due to its wide ecological tolerance, *Lantana camara* has a broad geographic distribution. It can thrive in various climates, habitats, and soil types, with precipitation amounts ranging from 750 to 5,000 mm per year and at altitudes up to 1,800 m a.s.l (Girish et al., 2019). *Lantana camara* prefers to grow in open, unshaded areas such as grasslands and foothills of tropical, subtropical, or degraded forests (Raghubanshi et al., 2005; Kandwal et al., 2009). In tropical, subtropical, and warm-temperate areas, it has developed into a rapidly spreading IAS with a high potential for damage (Hansda et al., 2024; Surya et al., 2024). The invasion of an ecosystem by *Lantana camara* is strongly facilitated by disturbances to natural environments, often caused by anthropogenic activities such as deforestation or agricultural practices (Shackleton et al., 2017). *Lantana camara* can become the dominant species in these disturbed areas, disrupting ecological succession and reducing biodiversity. Additionally, anthropogenic infrastructure, such as roadsides and railroads, are particularly susceptible to invasion (Raghubanshi et al., 2005; Walton, 2006). Due to its exceptional invasiveness and significant environmental and economic impacts, *Lantana camara* lists among the world's ten worst invasive plant species (Goyal and Sharma, 2015). Recent global-scale analyses highlight the species’ increasing invasion potential across continents attributed to climate change and anthropogenic pressures (Adhikari et al., 2024). In Rwanda, *Lantana camara* was initially introduced as an ornamental plant (Seburanga, 2015). However, once it escaped cultivation, it proliferated rapidly, beyond cultivation. Today, its spread has reached a concerning level in the country, as reported by Seburanga (2015). Despite extensive research on the control of *Lantana camara*, successful management has remained elusive in nearly all affected ecosystems (Gunasekara and Ranwala, 2018; Barahukwa et al., 2023). Identifying potential habitats and implementing strict monitoring schemes is therefore crucial to prevent new introductions or further range expansions (Qin et al., 2016; Kato-Noguchi and Kato, 2025). To address this challenge, species distribution models (SDMs) combined with earth observation technologies have recently emerged as promising tools. SDMs have gained increasing global attention for their ability to map the spatiotemporal distribution of IASs (Saranya et al., 2021).

While earth observation data, such as vegetation indices, show promising results in habitat suitability modeling, they remain underused, particularly in data-scarce regions like savannah ecosystems (Ahmed et al., 2020). However, remote sensing alone faces limitations in accurately detecting IAS in areas with dense canopies and high seasonal cloud coverage, conditions typical of savannah ecosystems (Purohit et al., 2019; Abdi et al., 2022). To overcome these challenges, combining remote sensing data with critical environmental predictors a SDM offers a more comprehensive solution for mapping IAS distribution. This integration enhances the ability to assess both the IAS’ current and potential spread, as well as identify their driving factors for invasion across spatial and temporal scales (Goncalves et al., 2014; Purohit et al., 2019; Adhikari et al., 2024).

Previous studies have demonstrated the effectiveness of multispectral satellite data in assessing habitat suitability for *Lantana camara* and other IAS (Lahoz-Monfort et al., 2010; Shirley et al., 2013; St-Louis et al., 2014). Optical and multispectral satellite sensors, such as Landsat-8 and Sentinel-2, have been widely used for mapping and discriminating IAS (Kandwal et al., 2009; Dube et al., 2020; Dube et al., 2022; Waititu et al., 2023). In a recent study, Mtyobila and Shoko (2024) successfully combined Sentinel-2 Multispectral Imager (MSI) data with the Random Forest (RF) classifier to model the spatial distribution of *Lantana camara* in a savannah ecosystem in South Africa.

Key predictors for *Lantana camara* habitat suitability include temperature, precipitation, land cover, elevation, and spectral data like the red edge (RE) and shortwave infrared (SWIR) bands (Vardien et al., 2012; Mungi et al., 2020; Mondal et al., 2021; Dube et al., 2022). Furthermore, soil characteristics (e.g., cation exchange capacity and nutrient content) have been linked to *Lantana camara* habitat suitability and can thus serve as effective predictors in modeling (Mungi et al., 2020; Mondal et al., 2021). Additionally, anthropogenic disturbance factors such as road networks and human settlement data have been identified as critical indicators of ecosystem perturbations, functioning as gateways for IAS (Akin-Fajiye and Akomolafe, 2021; Dube et al., 2022).

Despite advancements in IAS modeling with remote sensing data, most studies on *Lantana camara* dynamics remain geographically limited and are primarily conducted in India, Australia, or South Africa (Kandwal et al., 2009; Vardien et al., 2012; Dube et al., 2020; Mondal et al., 2021; Dube et al., 2022; Tarugara et al., 2022; Shiri et al., 2023). In contrast, Central and East Africa remain underrepresented in the literature despite growing concern over *Lantana camara*'s ecological impact in these regions (Vardien et al., 2012; Shackleton et al., 2017; Agaldo, 2019; Waititu et al., 2023). A recent field-based study in East Africa documented associations between *Lantana camara* and native vegetation, highlighting the species' disruptive ecological effects on savannah plant communities (Ssali et al., 2024).

This study assesses the changes in habitat suitability for *Lantana camara* over nine years (2015–2023) in the Akagera National Park in eastern Rwanda. Using open-access, high-resolution satellite imagery, we developed a comprehensive SDM within the Google Earth Engine (GEE) (Gorelick et al., 2017), integrating earth observation data with legacy environmental and anthropogenic parameters. A presence-only RF algorithm was used to predict the spatiotemporal distribution of *Lantana camara* across the park. This study introduces a novel and spatially explicit modeling framework for invasive species in Rwanda, where machine learning-based SDMs that integrate remote sensing and anthropogenic predictors remain underutilized despite growing invasion pressures (Seburanga, 2015; Eckert et al., 2020). By addressing key gaps in data-scarce ecosystems, our approach aims to support targeted conservation planning and establish a replicable method for IAS monitoring in East African savannahs.

## 2 Materials and methods

### 2.1 Study area

**Figure 1:**
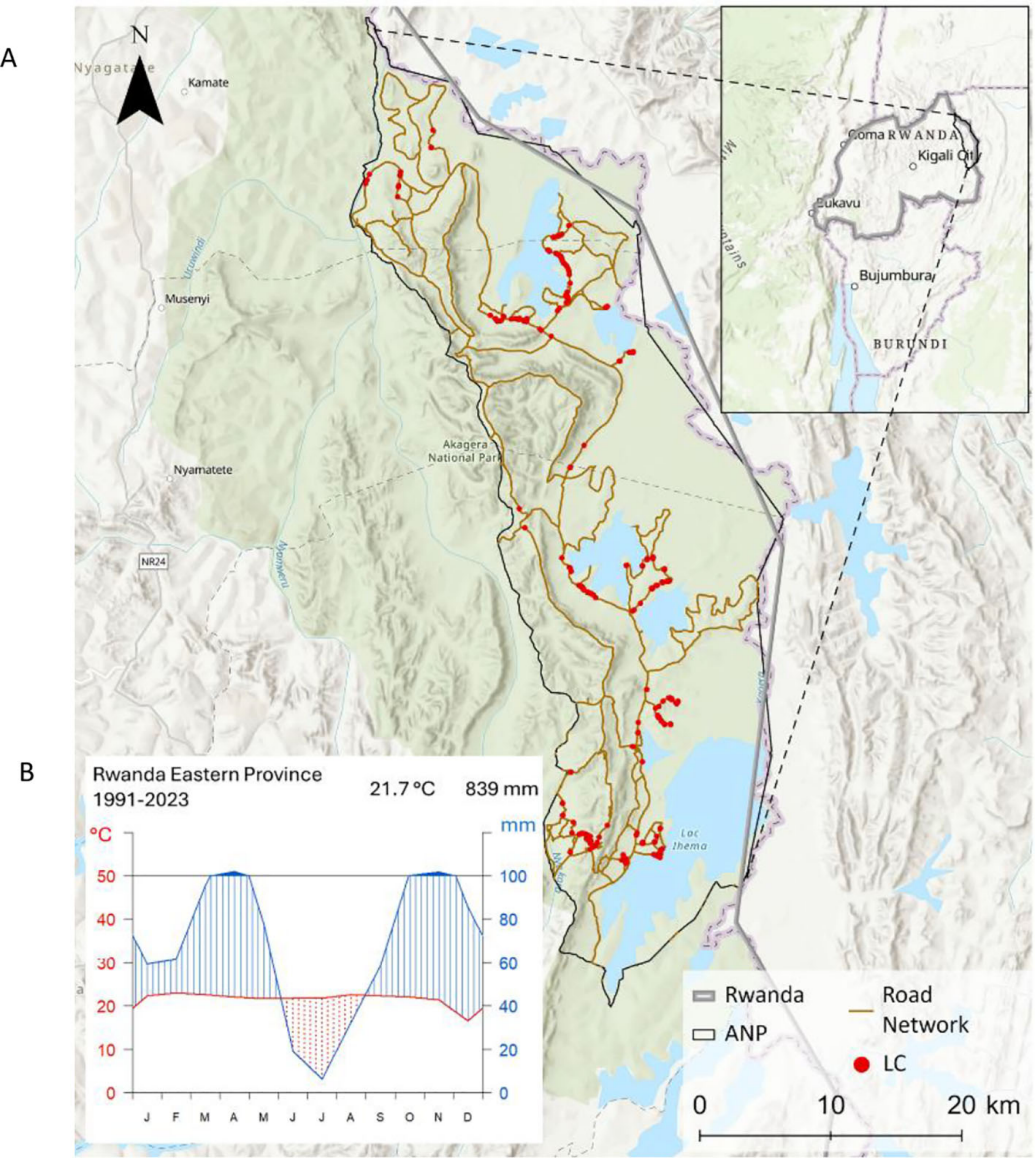
**(A)** Topographic map of the Akagera National Park in Eastern Rwanda, showing its road network (brown line) and ground-truth sampling locations of *Lantana camara* occurrences (red dots) (African Parks, 2015; Esri, 2024). **(B)** Climate diagram for the Eastern Province of Rwanda (1991–2023), based on ERA5 data, illustrating long-term mean monthly temperature (°C) and precipitation (mm) (Copernicus Climate Change Service, 2019).

Our study area is the Akagera National Park in Rwanda, East Africa (**Figure 1A**), located at 1°45′00″ S, 30°38′00″ E (Gatali and Wallin, 2015).

The elevation of Akagera National Park ranges from 1,286 m to 1,718 m a.s.l. The ecosystem within Akagera National Park is characterized by various habitat types, including swamps, grasslands, and dry and humid forests (Fishpool and Evans, 2001; Harding, 2009), exhibiting a high biodiversity. Rwanda’s temperate and humid climate is strongly shaped by its topography (**Figure 1B**). The western highlands are cooler and wetter, while the eastern lowlands are warmer and drier (Fishpool and Evans, 2001; IAEA, 2020). The bimodal precipitation regime includes a short dry season ( January–February), a long rainy season (March–June), a long dry season (June–September), and a short rainy season (October–December). The mean annual rainfall (1991–2023) in the Akagera National Park is 839 mm. The mean temperature ranges around 22°C (Copernicus Climate Change Service, 2019) (**Figure 1B**).

### 2.2 Reference dataset

The field campaign for sampling ground reference data took place over two weeks in November 2023, during the short rainy season in Akagera National Park. Due to operational constraints and safety considerations in the national park, our sampling was primarily restricted to accessible areas along the road network. Point data were sampled to map *Lantana camara* individuals and avoid mixing them with heterogenous thickets of other plants. The field survey yielded 647 *Lantana camara* presence points (**Figure 1A**).

This ground-truth dataset is of particular value due to the scarcity of regional presence data in global biodiversity repositories; for instance, the Global Biodiversity Information Facility (GBIF) lists only 21 geo-referenced *Lantana camara* records for the entire study area (GBIF.org, 2025).

### 2.3 Model setup

**Figure 2:**
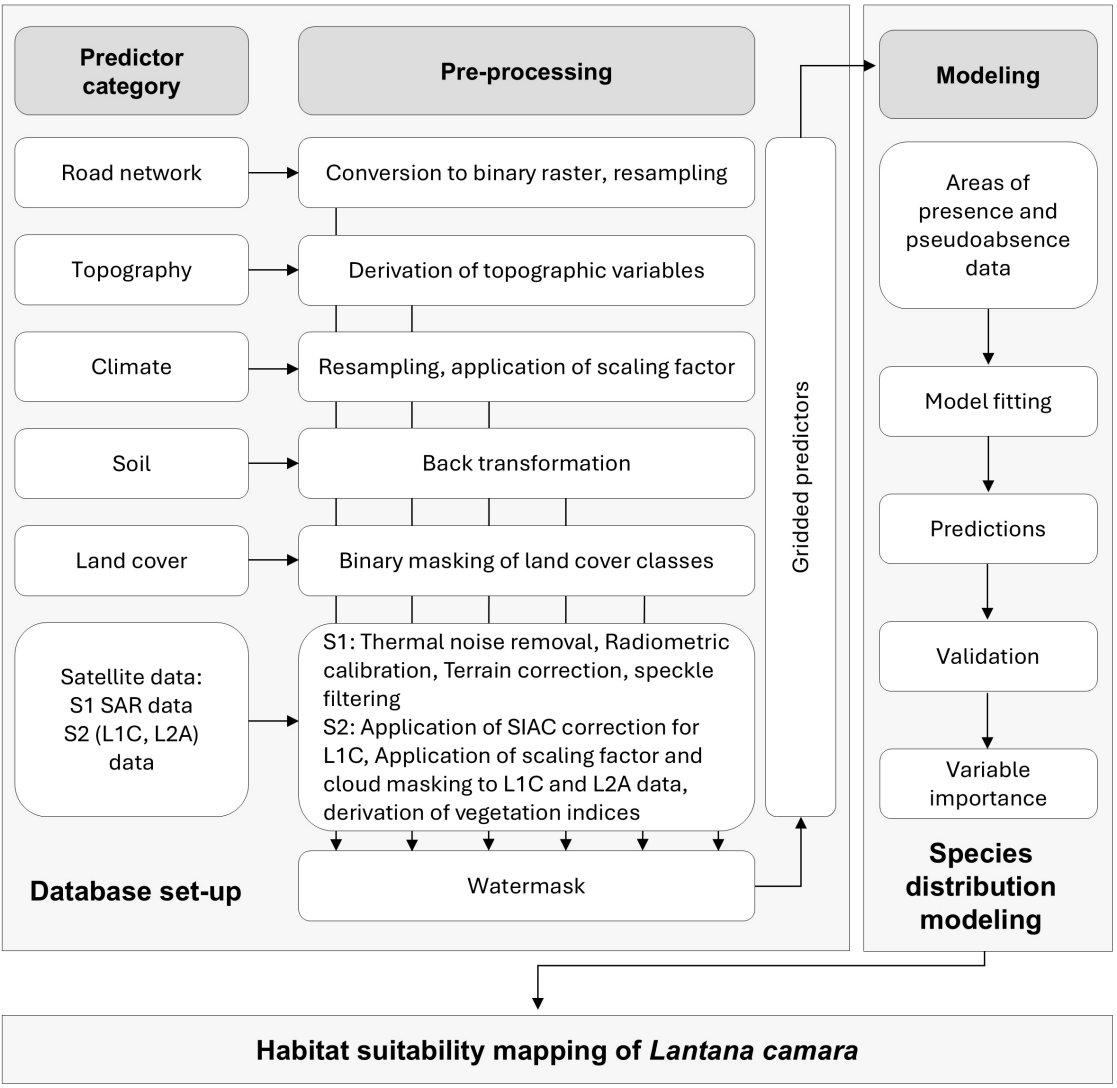
Study workflow to develop a species distribution model for *Lantana camara* habitat suitability modeling, showing the selected predictor categories and their required pre-processing steps.

The habitat suitability model was established in GEE at 30 m spatial resolution. Although Sentinel data at 10 and 20 m resolution were used for spectral analysis, 40 out of 52 predictor layers had a minimum native resolution of 30 m. Therefore, 30 m was selected as the standard resolution for the SDM to ensure consistency across input datasets. The methodological workflow involves selecting predictor categories, pre-processing and integrating individual predictors into the SDM, followed by model prediction using the RF algorithm and subsequent validation (**Figure 2**). Initially, to reduce the impact of sampling bias from data aggregation, *Lantana camara* location data were thinned to one randomly selected occurrence per pixel at the 30 m cell size in GEE (Holmes, 2020; Bracken et al., 2022). This process reduced the original 647 *Lantana camara* presence points to 387.

### 2.4 Predictor categories

*Lantana camara* invasiveness and habitat suitability are affected by various ecological and anthropogenic factors. Therefore, the SDM integrates a diverse set of predictors, selected based on the ecological traits of *Lantana camaras* and their consistent identification as relevant factors in previous studies (Raghubanshi et al., 2005; Mandal and Joshi, 2015; Wronski et al., 2017; Dube et al., 2020). Seven overarching predictor categories were chosen for model development, encompassing a total of 52 gridded predictors after pre-processing (**Figure 2**; **Table 1**). Among these, only a subset of predictors varied over time, including climatic variables, Land Cover Classifications (LCC), Sentinel-2 spectral data, and Sentinel-1 Synthetic Aperture Radar (SAR) backscatter. In contrast, topographic, soil, and road network predictors were considered time-invariant and included as constant layers in the SDM.

**Table 1:** List of predictor categories and derived predictors with specifications on their unit, polarization or central wavelength, and the name of the gridded predictors as implemented into the species distribution modeling framework.

Predictor category	Predictor; unit/polarization/central wavelength	Gridded predictor name	Data source
Road network	Roads	Roads	(African Parks, 2024)
Topography	Aspect; °	Aspect	(Farr et al., 2007)
	Elevation; m a.s.l.	Elevation	
	Slope; °	Slope	
	Topographic position index; -	TPI	
	Topographic wetness index; -	TWI	
Climate	Actual evapotranspiration; mm	AET	(Abatzoglou et al., 2018)
	Potential evapotranspiration; mm	PET	
	Maximum temperature; °C	TMMX	
	Minimum temperature; °C	TMMN	
	Precipitation; mm	P	
	Soil moisture; mm	SOIL	
Soil	Topsoil sand content; %	Sand_TS	(Hengl et al., 2021)
	Subsoil sand content; %	Sand_TS	
	Topsoil clay content; %	Clay_TS	
	Subsoil clay content; %	Clay_SS	
	Topsoil organic carbon; g kg^-1^	C_TS	
	Subsoil organic carbon; g kg^-1^	C_SS	
	topsoil effective cation exchange capacity; cmol+ kg^-1^	ECC_TS	
	subsoil effective cation exchange capacity, cmol+ kg^-1^	ECC_TS	
	topsoil nitrogen; g kg^-1^	N_TS	
	subsoil nitrogen; g kg^-1^	N_SS	
	topsoil phosphorus; ppm	P_TS	
	subsoil phosphorus; ppm	P_TS	
	topsoil pH	pH_TS	
	subsoil pH	pH_SS	
Land cover	class ‘Bare ground’	Bare_Ground	see section 1.4.5
	class ‘Bushland’	Bushland	
	class ‘Forest’	Forest	
	class ‘Grassland’	Grassland	
	class ‘Wetland’	Wetland	
	class ‘Woodland’	Woodland	
S1 SAR backscatter	VH, σ°	VH	(ESA, 2024a)
	VV, σ°	VV	
S2 spectral data	Blue band; 492.4 nm (S2A), 492.1 nm (S2B)	Blue	(ESA, 2024b, 2024c)
	Green band; 559.8 nm (S2A), 559.0 nm (S2B)	Green	
	Red band; 664.6 nm (S2A), 665.0 nm (S2B)	Red	
	Red edge band 1; 704.1 nm (S2A), 703.8 nm (S2B)	RE1	
	Red edge band 2; 740.5 nm (S2A), 739.1 nm (S2B)	RE2	
	Red edge band 3; 782.8 nm (S2A), 779.7 nm (S2B)	RE3	
	Near-infrared band; 832.8 nm (S2A), 833.0 nm (S2B)	NIR	
	Narrow Near-infrared band; 864.7 nm (S2A), 864.0 nm (S2B)	RE4	
	Shortwave infrared band 1; 1613.7 nm (S2A), 1610.4 nm (S2B)	SWIR1	
	Shortwave infrared band 2; 2202.4 nm (S2A), 2185.7 nm (S2B)	SWIR2	
S2-based vegetation indices	Canopy Chlorophyll Content Index; -	CCCI	(Person, 1972; Rouse et al., 1973; Huete, 1988; Gitelson et al., 1996; Barnes et al., 2000; Broge and Leblanc, 2001)
	Green Normalized Difference Vegetation Index; -	GNDVI	
	Normalized Difference Red Edge Index; -	NDRE	
	Normalized Difference Vegetation Index;	NDVI	
	Ratio vegetation index; -	RVI	
	Soil-adjusted Vegetation Index, L Factor 0.5; -	SAVI_L05	
	Soil-adjusted Vegetation Index, L Factor 1.0; -	SAVI_L1	
	Transformed Vegetation Index; -	TVI	

- = dimensionless.

To ensure consistency across datasets, all predictors were water-masked during pre-processing, (**Figure 2**).

#### 2.4.1 Road network

As anthropogenic structures have been shown to impact the distribution of *Lantana camara*, the park's road network was included as a constant predictor (Raghubanshi et al., 2005; Walton, 2006; Mondal et al., 2022). The data, initially provided by African Parks (2024) in vector format, were buffered by 50 m on each side and subsequently converted to a binary raster format at 30 m spatial resolution (**Figure 2**).

#### 2.4.2 Topography

Topographic parameters can affect *Lantana camara* invasiveness by impacting environmental conditions like net radiation and soil moisture distribution (Dube et al., 2022; Mtyobila and Shoko, 2024). Alongside elevation, aspect, and slope, the Topographic Position Index (TPI) and Topographic Wetness Index (TWI) were included in the model for additional topographic information, as demonstrated by Dube et al. (2022) and Mtyobila and Shoko (2024). All topographic predictors used in this study were computed in GEE based on the NASA SRTM Digital Elevation dataset with a spatial resolution of 30 m (Farr et al., 2007) and were treated as constant inputs in the model (**Figure 2**)

#### 2.4.3 Climate

Precipitation, temperature, evapotranspiration, and soil moisture data were used as climatic predictors in the model (**Table 1**). We acquired the data from TerraClimate at a spatial resolution of approximately 4.6 km (Abatzoglou et al., 2018). To ensure compatibility with other predictors used in the SDM, we resampled the climate layers in RStudio to 30 m using bilinear interpolation (Hijmans and Van Etten, 2012). We then uploaded the processed layers to GEE for further processing. In addition, the appropriate scaling factor, as specified by Abatzoglou et al. (2018), was applied to all data in GEE. The climate data were summarized as 11-month medians, encompassing the 10 months before the study period (January–October) and each year's November, to account for the entire vegetation period (**Figure 2**).

#### 2.4.4 Soil

Given the reciprocal relationship between *Lantana camara* and soil properties (Mondal et al., 2022), seven soil parameters were incorporated as static predictors into the SDM (**Table 1**). These predictors were selected as they have previously been linked to *Lantana camara* habitat distribution (Vardien et al., 2012; Mungi et al., 2020; Mondal et al., 2022). Both the topsoil (predicted mean at 0–20 cm depth) and subsoil (predicted mean at 20–50 cm depth) properties were included as individual predictors (**Table 1**). The data, taken from *Innovative Solutions for Decision Agriculture Ltd* (iSDA; ) at a 30 m resolution, required individual back-transformation to their respective units as indicated by Hengl et al. (2021) (**Figure 2**). Soil texture (e.g., clay content) and chemical properties (e.g., nitrogen content) were selected for the analysis. Additionally, the effective cation exchange capacity was included as a parameter for soil fertility (Vardien et al., 2012; Mungi et al., 2020; Mondal et al., 2022).

#### 2.4.5 Land cover

The 30 m spatial resolution LCC dataset used in this study was derived from a long-term (1984 to 2023) time series that was constructed as part of a land cover pre-survey for this work. Median-aggregated Landsat-5, Landsat-7, and Landsat-8 imagery and ground-truth data from Akagera National Park sampled by us in the years 2020 and 2023 were utilized to develop the respective LCC. The classification model was extrapolated based on a single high-accuracy classification of the 2020 median image to address the temporal limitation of reliable ground-truthing. To find an optimal set of predictors for this, subsets were formed. All combinations were tested with a ten-fold cross-validation (Wong and Yeh, 2020). Synthetic training points were incorporated to ensure an adequate number of training points for accurate land cover predictions. The machine learning-based approach enabled the reconstruction of retrospective land cover classifications, providing a robust dataset despite limited ground-truth data challenges. The LCC consists of seven classes (**Table 1**). All classes were included in the SDM except for water, which was excluded due to the addition of a water mask to all predictors during pre-processing (**Figure 2**). Due to a notable cloud coverage in the years 2015 and 2018, a complete coverage of our study area for the LCC data was not achieved. To mitigate this issue, data from adjacent years were used as substitutes: 2016 data for 2015 and 2017 data for 2018. For the three years 2021-2023, the 2020 LCC was employed in the SDM, as more recent data at a suitable spatial resolution were unavailable for the study area.

#### 2.4.6 S1 SAR backscatter

SAR data from the Sentinel-1A (S1A) satellite were included as predictors in the SDM. Specifically, Level-1 Ground Range Detected C-band data with a 10 m spatial resolution. For this study, dual-polarization measurements were used, in the interferometric wide swath (IW) acquisition mode with vertical launch and vertical reception (VV) as well as vertical launch and horizontal reception (VH) (El Hajj et al., 2019; Vreugdenhil et al., 2020). These measurements were chosen due to the demonstrated co- and cross-polarized backscatter sensitivity to vegetation (El Hajj et al., 2019; Vreugdenhil et al., 2020). The backscatter values from S1A data were obtained in sigma nought (σ°) units. Each scene underwent thermal noise removal, radiometric calibration, and terrain correction using SRTM 30 within the S1 Toolbox (ESA, 2024a). A refined Lee speckle filter was also applied to reduce noise and improve image quality (Yommy et al., 2015). During this step, the backscatter values were processed in natural units before being converted back to decibels for subsequent analysis (Gandhi, 2021) (**Figure 2**). Although SAR has been underutilized in SDM development and *Lantana camara* detection, its unique capabilities offer notable advantages. SAR can operate independently of cloud cover and illumination, allowing for data acquisition both during the day and at night (Rajah et al., 2019).

#### 2.4.7 S2 spectral data

For spectral imagery, Sentinel-2 (S2) Level-2A (L2A) MSI data from 2019-2023 (ESA, 2024c) were used, while for 2015–2018, only Level-1C (L1C) data (ESA, 2024b) were available. For the L1C data atmospheric correction was performed in GEE using the Sensor Invariant Atmospheric Correction (SIAC) package by Yin et al. (2022), converting TOA imagery to surface reflectance values. All S2 data underwent additional cloud masking in GEE using the SCL band for L2A data (2019–2023) and the cloud probability dataset for L1C data (2015–2018) (ESA, 2024b, ESA, 2024c). Data gaps in the imagery due to high cloud cover and shadows were filled using composites from adjacent years based on the Earth Engine Data Catalog (2024) to obtain complete coverage of the study area (**Table 2**; **Figure 2**).

**Table 2:** Overview of the SAR (Sentinel-1) and multispectral (Sentinel-2) data used in the species distribution model for the study period, including selected time periods for SAR composites, cloud-masked multispectral composites, and the corresponding replacement composites.

Data	Data specification	2015	2016	2017	2018	2019	2020	2021	2022	2023
Sentinel-1 SAR	Period	01.11.2015 – 30.12.2015	01.11.2016 – 30.11.2016	01.11.2017 – 30.11.2017	01.11.2018 – 30.11.2018	01.11.2019 – 30.11.2019	01.11.2020 – 30.11.2020	01.11.2021 – 30.11.2021	01.11.2022 – 30.11.2022	01.11.2023 – 30.11.2023
	Number of Images (VV and VH Polarization)	3	3	14	12	18	17	13	13	10
Sentinel-2 (MSI)	Level	L1C				L2A					
	Period	01.11.2015 – 30.11.2015	01.11.2016 – 30.11.2016	01.11.2017 – 30.11.2017	01.11.2018 – 30.11.2018	01.11.2019 – 30.11.2019	01.11.2020 – 30.11.2020	01.11.2021 – 30.11.2021	01.11.2022 – 30.11.2022	01.11.2023 – 30.11.2023
	Number of Cloudy Scenes	8	20	17	6	6	6	4	13	7
	Replacement Period	01.09.2014 – 30.12.2014	01.09.2015 – 30.12.2015	01.09.2016 – 30.12.2016	01.09.2017 – 30.12.2017	01.10.2019 – 30.12.2019	01.10.2020 – 30.12.2020	01.10.2021 – 30.12.2021	01.10.2022 – 30.12.2022	01.10.2023 – 30.12.2023
	Number of Replacement Scenes	76	48	121	45	10	17	7	6	8

In addition to the spectral bands implemented in the SDM, seven different vegetation indices (**Table 3**) were generated from the S2 images. The indices were selected based on their general vegetation discrimination performance and documented use in similar studies (Kandwal et al., 2009; Dube et al., 2022; Waititu et al., 2023).

**Table 3:** Utilized vegetation indices as predictors in the species distribution model.

Index	Formula	Reference
*Canopy Chlorophyll Content Index*	$CCCI=\frac{\left(\frac{NIR-REDedge}{NIR+REDedge}\right)}{\left(\frac{NIR-RED}{NIR+RED}\right)}$	*(* Barnes et al., 2000 *)*
*Green Normalized Difference Vegetation Index*	$GNDVI=\frac{NIR-GREEN}{NIR+GREEN}$	*(* Gitelson et al., 1996 *)*
*Normalized Difference Red Edge*	$NDRE=\frac{NIR-REdedge}{NIR+Rededge}$	*(* Barnes et al., 2000 *)*
*Normalized Difference Vegetation Index*	$NDVI=\frac{NIR-RED}{NIR+RED}$	(Rouse et al., 1973)
*Ratio Vegetation Index*	$RVI=\frac{NIR}{RED}$	(Person, 1972)
*Soil-Adjusted Vegetation Index*	$SAVI=\frac{\left(NIR-RED\right)}{\left(NIR+RED+L\right)}\times \left(1+L\right)\;\;\;$	*(* Huete, 1988 *)*
*Transformed Vegetation Index*	$TVI=\;\sqrt{\left(NDVI\right)+0.5}$	*(* Broge and Leblanc, 2001 *)*

### 2.5 Prediction of pseudo-absences and model fitting

The final 52 pre-processed gridded predictors were compiled into a multi-band raster image with 30 m spatial resolution, each band representing a different predictor, providing the foundation for correlating environmental and earth observation data with *Lantana camara* presence points (**Figure 2**).

The developed SDM used RF classification trees to create predictive distribution maps from presence-only data. The algorithm was chosen for its many advantages, like its robustness with high-dimensional data, ability to handle multicollinearity, and strong predictive performance (Breimann, 2001; Zhang et al., 2019; Adhikari et al., 2024).

Given the lack of true absence data, pseudo-absences were generated using a two-step profiling approach: masking presence locations to avoid overlap and applying k-means clustering to identify environmentally distinct areas (Barbet-Massin et al., 2012; Sillero et al., 2021).

A spatial block cross-validation technique with ten iterations was applied, partitioning data into 70% training and 30% validation sets. Equal numbers of pseudo-absences and presence points were generated per block to ensure dataset balance, which has been argued to perform better with machine-learning algorithms (Barbet-Massin et al., 2012; Crego et al., 2022).

Each training dataset was then fitted using an RF classifier (Mi et al., 2017; Google Earth Engine, 2023). The output included classifications of presence probabilities, presence-absence data, and training and testing partitions. A final habitat suitability map was generated by averaging the presence probability outputs from all ten iterations. Additionally, a binary presence-absence map was created using a majority vote across the ten binary model outputs, which was then used to quantify the potential suitable habitat for *Lantana camara* throughout the study period. Moreover, the predictors' individual variable importances were extracted from the presence probability classifier. For the year 2023 the implemented list of predictors was narrowed down to the seven most impactful predictors based on a threshold of 3.5% for relative contribution to the SDM. This subset was then used to rerun the SDM for a more detailed analysis for 2023 (Crego et al., 2022; Crego et al., 2023).

The resulting Habitat Suitability Index (HSI) maps were further utilized to create pixel change maps for temporal comparability. This additional analysis facilitated a more comprehensive examination of habitat dynamics throughout the study period.

### 2.6 Validation

Model performance was assessed using Area Under the Receiver Operating Characteristic Curve (AUC_ROC_), the Area Under the Precision-Recall Curve (AUC_PR_), sensitivity (true positive rate), and specificity (true negative rate) metrics applied to the validation data across ten model iterations (Crego et al., 2022). AUC_ROC_ and AUC_PR_ are threshold-independent metrics widely used in ecological niche modeling to assess the ability of a model to distinguish between presence and absence sites (Valavi et al., 2021; Dastres et al., 2025).

## 3 Results

### 3.1 Spatio-temporal dynamics of habitat suitability

**Figure 3:**
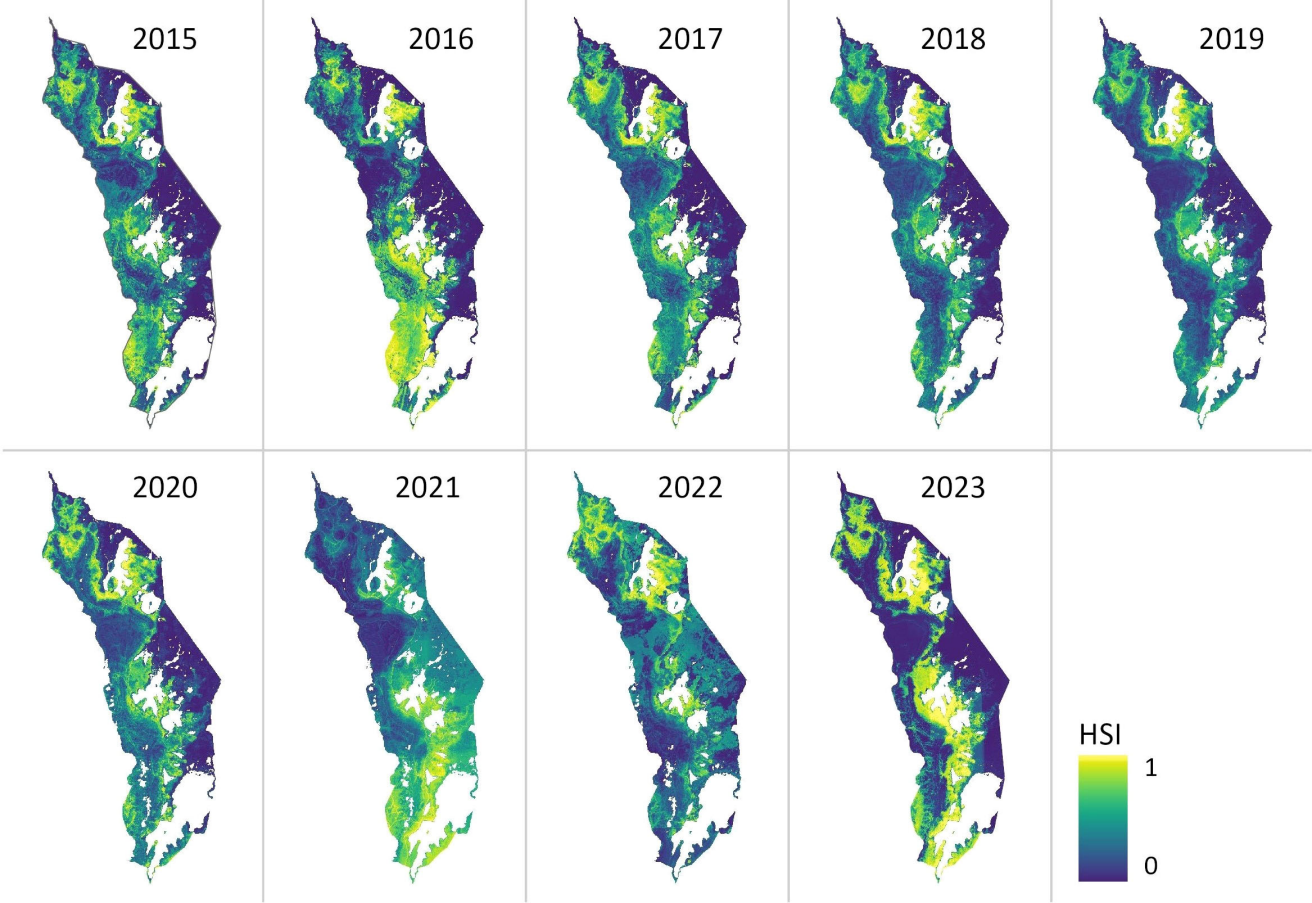
Predicted habitat suitability index (HSI) for *LantanaLantana camara* in AkageraAkagera National Park from 2015 to 2023. The likelihood of the occurrence of *LantanaLantana camara* is estimated on a scale from 0-1. Zero (dark blue) indicating the lowest and one (yellow) the highest habitat suitability.

The annual habitat suitability maps generated by the SDM reveal substantial areas of suitable habitat for *Lantana camara* throughout Akagera National Park during the 2015–2023 study period (**Figure 3**). Notably, suitability patterns show considerable spatial variation between years. The years 2019 and 2018 show the smallest area of suitable *Lantana camara* habitat, whereas 2016 and 2023 exhibit the largest area (**Figure 3**). In 2023, 33% of the study area was predicted to be suitable for infestation. As expected, the predicted suitable habitat is closely spatially related to the *Lantana camara* sampling sites (**Figure 1**). Furthermore, while most observed years show a consistent pattern of predicted habitat concentrated in the central areas of Akagera National Park, some years, notably 2016 and 2021, stand out with significant deviations, featuring extensive hotspots in the park's southern region. Additionally, 2023 is noteworthy for the substantial expansion of the overall potential habitat area (**Figure 3**).

**Figure 4:**
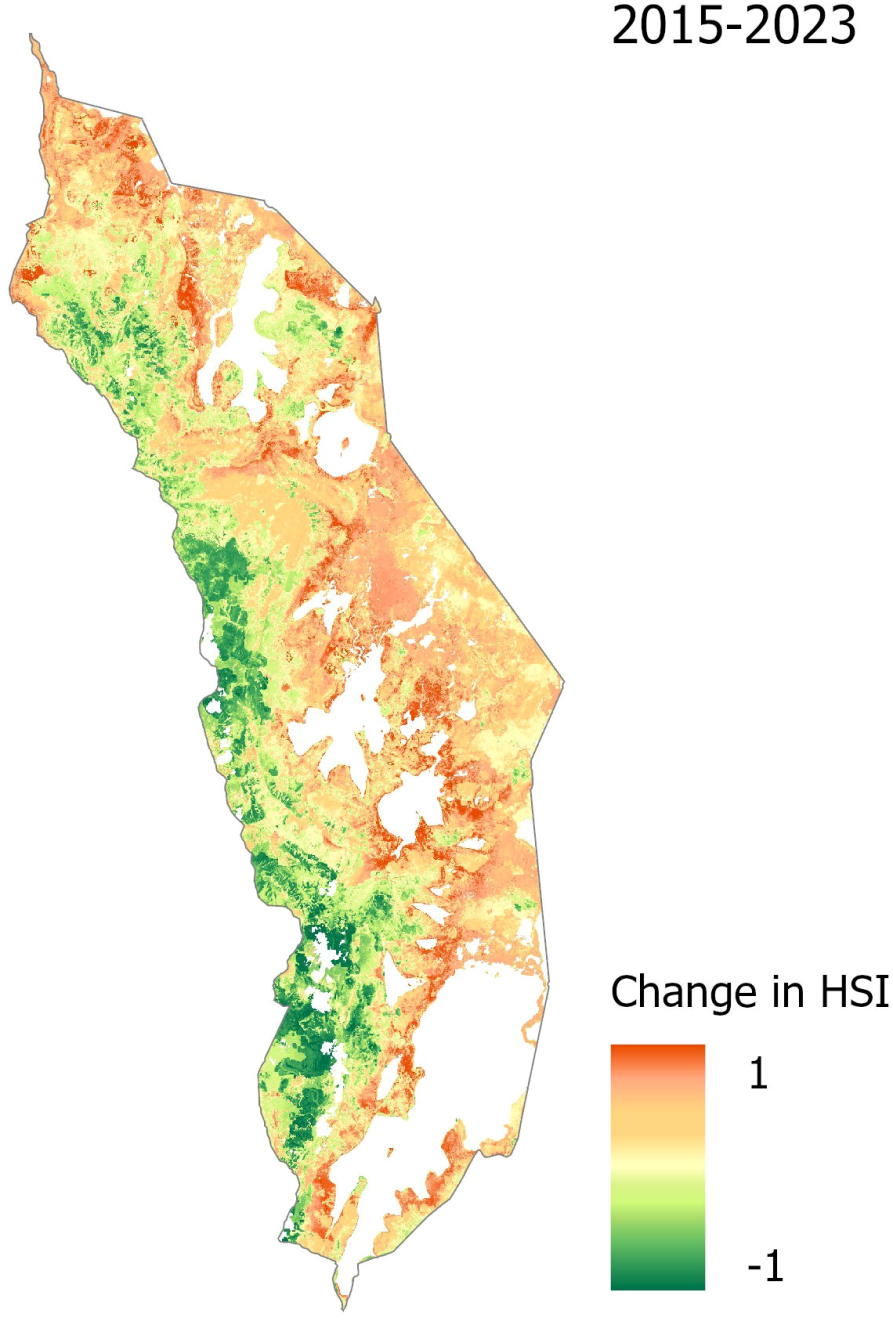
Change detection of habitat suitability for *Lantana camara* in Akagera National Park from 2015 to 2023. Red indicates a positive change in the habitat suitability index (HSI), thus an increase in habitat suitability for *Lantana camara*, while green denotes a decrease in habitat suitability over the study period.

A change detection analysis of the annual HSI maps further highlights dynamic changes in suitable habitat across the study period (**Figure 4**, **Table 4**). The total area classified as suitable varied considerably from year to year, with the lowest extent in 2018 (163.79 km²) and the highest in 2023 (364.85 km²) (**Table 4**). While interannual fluctuations are evident, a general upward trend in habitat suitability is observed over time. The central park region consistently exhibits increased suitability, while the northeastern zone shows a marked expansion of favorable conditions. In contrast, the southwestern region of the park demonstrates a decline in suitable habitat (**Figure 4**).

**Table 4:** Annual change in predicted suitable habitat area for *Lantana camara* in Akagera National Park (2015–2023).

Year	Suitable Habitat (km²)	Annual Change (km²)
2015	230,01	–
2016	295.18	+65.17
2017	206.05	-89.13
2018	196.05	-10.00
2019	163.79	-32.26
2020	210.19	+46.40
2021	281.87	+71.68
2022	225.90	-55.97
2023	364.85	+138.95

### 3.2 Performance of individual predictors

**Figure 5:**
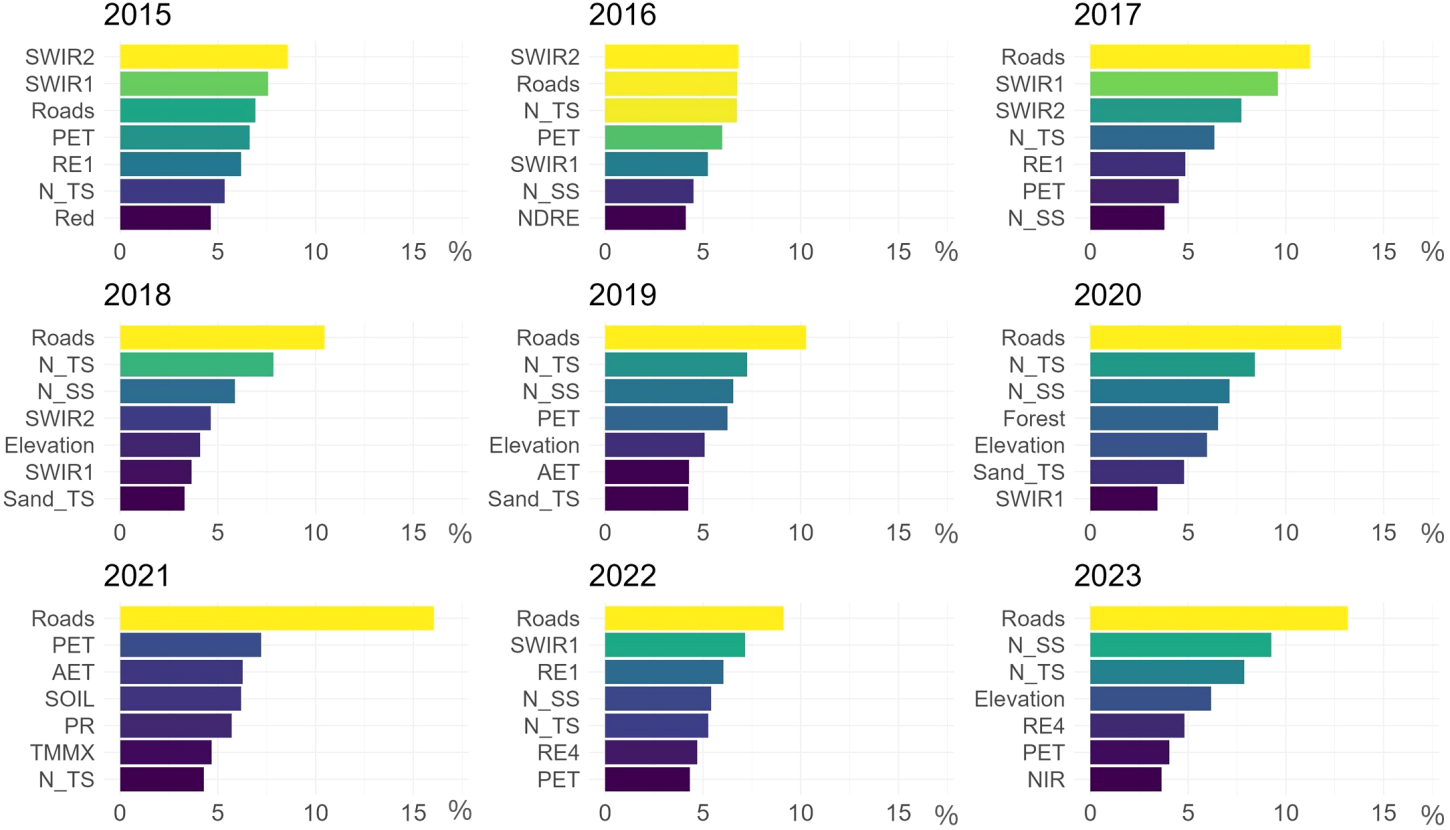
Relative contribution (%) of the seven most important predictors for each observed year, as determined by the Random Forest classification. Higher percentages (yellow to green shades) indicate higher variable importance and hence better prediction capabilities.

This study assessed the performance of predictors integrated into the SDM based on their effectiveness in predicting *Lantana camara* habitat suitability in Akagera National Park. The variable importances, derived from the presence probability classifier, indicate each predictor's ability to distinguish suitable from unsuitable *Lantana camara* habitat within the training dataset (**Figure 5**). Throughout the study period, the predictor Roads emerges as the predominant predictor. Except for 2015 and 2016, where the SWIR2 spectral band takes precedence, Roads can be identified as the most influential. Notably, in 2021, its influence is significantly stronger than in other years (**Figure 5**).

The pedological predictors, especially N_TS and N_SS, also contribute significantly. The nitrogen topsoil predictor fluctuates between 4.3% relative contribution in 2021 and 8.4% in 2020, while subsoil ranges from 3.8% in 2017 to 9.3% in 2023, highlighting the continued relevance of this soil nutrient in the SDM (**Figure 5**).

Among climatic factors, PET and temperature-related predictors (TMMX, TMMN) have the strongest influence in most years. In 2021, climatic predictors AET, PET, PR, SOIL, and TMMX are the most influential after Roads (**Figure 5**).

Elevation consistently is the most relevant topographic predictor across all examined years. The spectral bands, such as BLUE, GREEN, and RED demonstrate moderate influence on model predictions, while SWIR and RE bands are significantly influential in most analyzed years. In 2023, the NIR band also shows high predictive capabilities (**Figure 5**). The spectral vegetation indices evaluated for their impact on predicted habitat suitability show varied contributions, primarily on the lower to moderate end, ranging from 0.5% for RVI in 2020 to 4.1% for NDRE in 2016 (**Figure 5**).

The SAR-derived predictors VV and VH have a minor influence on the SDM for *Lantana camara*.

Land cover classes had minimal impact throughout the study period compared to other predictors. 2020 exhibited noticeably higher variable importances for Forest, Wetland and Woodland, with Forest ranking as the fourth strongest predictor (**Figure 5**).

In summary, the road network, soil nitrogen content, elevation, SWIR and RE spectral bands, and evapotranspiration data emerged as the most influential predictors in the model for *Lantana camara* in Akagera National Park.

**Figure 6:**
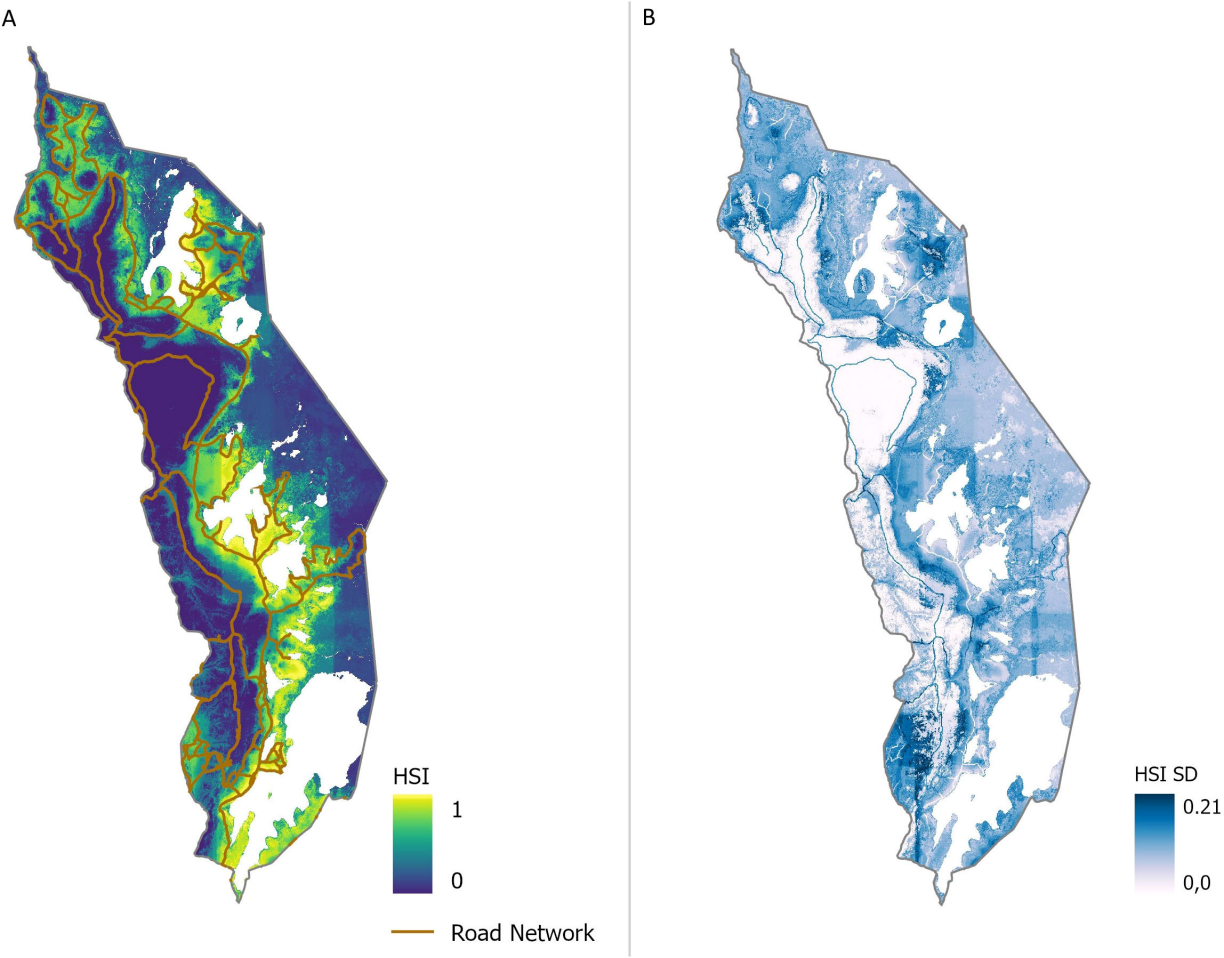
Mean habitat suitability index (HSI) results **(A)** and corresponding standard deviations (SD) **(B)** of HSI of additional model run with predictor subset for 2023. High HSI is symbolized in yellow, moderate HSI in green shades, and low HSI in blue. SDs are gradually symbolized from white to blue.

For 2023, the year of the ground-truth sampling, the seven most influential predictors in the SDM iterations, each with a relative contribution greater than 3.5%, were used for an additional model run for 2023, yielding statistically satisfactory results with a mean AUC_ROC_ of 0.98 and a mean AUC_PR_ of 0.78 (**Figure 6A**). The subset model provides a more apparent distinction between low and high habitat suitability compared to the comprehensive model that included all 52 predictors (**Figure 3**). High HSI values for the road network are evident in **Figure 6A**, reflecting its significant predictor impact. Most areas of Akagera National Park showed consistent predictions with minimal variation across model runs, except for the southwestern area and the road network, which exhibited relatively high SDs (0.21). Generally, areas of high habitat suitability displayed larger variability in model runs than those with low HSI values (**Figures 6A, B**).

### 3.3 Model performance

**Figure 7:**
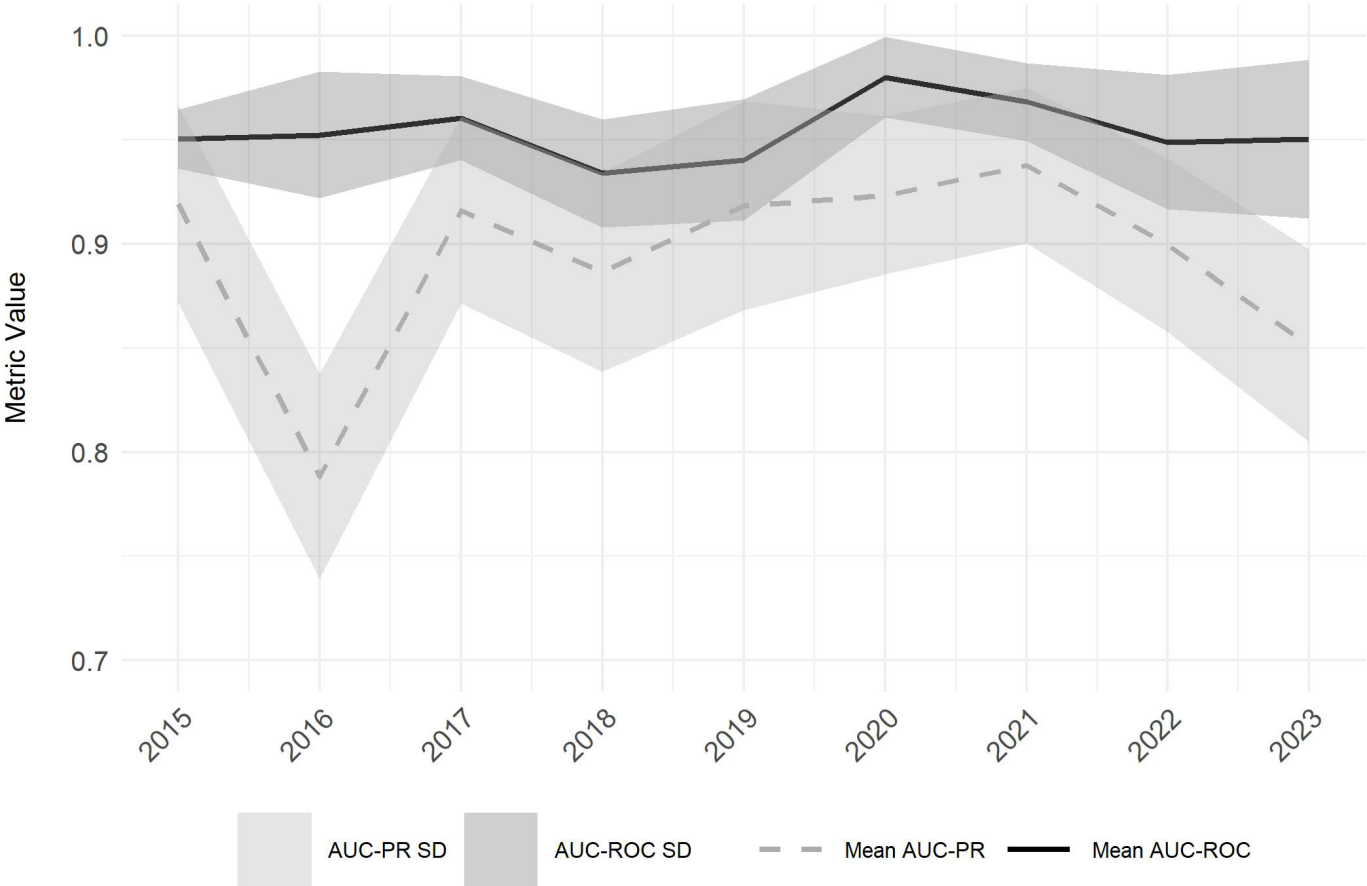
Mean Area Under the Precision-Recall Curve (AUC_PR_) (dashed grey line) and Mean Area Under the Receiver Operating Characteristic Curve (AUC_ROC_) (black line) values with their corresponding standard deviations (SD) (AUC_PR_: light grey shaded area, AUC_ROC_: dark grey shaded area) across all model iterations for the observed period (2015 to 2023).

Following the spatial predictions, we evaluated how reliably the SDM performs across years. Model results are consistent, with low standard deviations (SD) among model predictions of the individual years. The SDM also exhibits high predictive power with a mean AUC_ROC_ for the nine years analyzed ranging from 0.93 (2018) to 0.98 (2020), with SDs ranging from 0.01 to 0.04. Mean AUC_PR_ values are slightly lower on average and range from 0.79 (2016) to 0.94 (2021), with SDs of 0.04 and 0.05 (**Figure 7**).

The AUC_PR_ displays more variability over time compared to the AUC_ROC_, particularly evident in 2016, when the AUC_PR_ dropped to 0.79, marking a significant decline compared to other years. This decrease corresponds with the qualitative findings presented in **Figure 3**. In contrast, the AUC_ROC_ metric remains relatively stable throughout this period, showing no significant deviations (**Figure 7**).

Sensitivity and specificity metrics were also calculated, displaying more pronounced fluctuations over time and higher SDs on average. Mean sensitivity ranges from 0.89 (2015) to 0.94 (2017) with SDs of 0.03 - 0.08, and mean specificity values range from 0.81 (2018) to 0.94 (2020) with SDs ranging from 0.05 to 0.1 for the 10 model iterations.

## 4 Discussion

### 4.1 Invasion dynamics and key predictors of habitat suitability

The results indicate high predictive model performance, with AUC_ROC_ values ranging from 0.93 to 0.98 and AUC_PR_ values from 0.79 to 0.94 (**Figure 4**). The high AUC_ROC_ values demonstrate the model's capacity to discriminate between presence and absence sites of *Lantana camara*, while AUC_PR_ values suggest effective prediction of presence data (Valavi et al., 2022; Crego et al., 2023). The observed slight discrepancy between the two metrics may be due to the narrow distribution of sampled *Lantana camara* points in Akagera National Park, indicating that the AUC_ROC_ may assess model performances overly optimistic (Sofaer et al., 2019). Despite fluctuations in AUC_PR_, particularly in 2016, AUC_ROC_ values remained stable, indicating the model's robustness in identifying suitable habitats (**Figure 4**).

Throughout the study the road network emerged as the most influential predictor of habitat suitability, as additionally confirmed by subset calculations for 2023 (**Figure 6**). This finding aligns with previous studies, such as those by Dube et al. (2022) and Mondal et al. (2022), which emphasize higher invasion potential in disturbed areas like roadsides. Akagera National Park conservationists further substantiate these observations, identifying a prevalence of *Lantana camara* growth near roads and human settlements. In a related study by Shiri et al. (2023), increased *Lantana camara* invasion near roads was linked to disturbance from road maintenance activities, and the potential for seed dispersal via car tires, vehicle movement and livestock. Our results suggest that similar mechanisms may be influencing suitability patterns in Akagera National Park, where unpaved roads and associated land disturbances may be facilitating spread along edges and corridors (Mondal et al., 2022; Shiri et al., 2023).

Two other significant predictors in the study were nitrogen levels in both topsoil and subsoil, highlighting soil nutrients' importance in predicting *Lantana camara* distribution. Previous research supports these findings, showing that understory IAS can modify soil parameters, including nutrient inputs and cycling (Mandal and Joshi, 2015; Shiri et al., 2023). Similar patterns observed in woodland ecosystems indicate that the IAS tends to occur more frequently in areas with elevated nitrogen availability, where its efficient nutrient uptake may provide a competitive advantage Shiri et al., 2023).

Of the S2 spectral bands, the SWIR and RE spectral bands yielded high variable importances in most years. These findings align with Dube et al. (2020), who reported that SWIR wavelengths are among the most successful spectral bands in discriminating *Lantana camara* from different land cover types due to their sensitivity to species chlorophyll concentration and leaf water content (Clark et al., 2005). The RE bands, capable of capturing plant-specific reflectance characteristics and facilitating species discrimination, also yielded high variable importances throughout the study period. These bands focus on chlorophyll absorption and internal reflection, enabling the detection of subtle differences in foliar chemicals or chlorophyll concentrations (Mutanga and Skidmore, 2004; Dube et al., 2020). This enhances the spectral characterization of species, improving discrimination accuracy. It can be hypothesized that *Lantana camara* exhibits distinct chlorophyll concentrations compared to other vegetation types in Akagera National Park, aiding its identification via spectral imagery (Dube et al., 2020).

Elevation consistently ranked among the top predictors, reflecting its strong influence on the spatial distribution of *Lantana camara*, particularly in lower-lying regions. (**Figure 5**). Comparing the HSI data with **Figure 1** shows that habitat suitability declines at higher altitudes, especially in the park's western region. These findings align with the literature, which notes that *Lantana camara* thrives below 1800 m a.s.l (Girish et al., 2019). Elevation can also affect distribution due to its correlation with climatic factors, causing temperature and soil variations along the elevation gradient (Mtyobila and Shoko, 2024). This result is consistent with other studies reporting a strong dependency of *Lantana camara* habitat suitability on terrain parameters (Mungi et al., 2018; Dube et al., 2022; Mondal et al., 2022).

The land cover classes showed low to moderate variable importances throughout the study period. It should be considered when interpreting the results that the LCC are approximations. However, given their relatively small influence on the final model, potential inaccuracies will likely have a minimal impact. The Wetlands class was among the more influential LCC classes, as *Lantana camara* prefers boggy or hydromorphic soils and is sensitive to aridity, thriving in high water availability areas (Raghubanshi et al., 2005; Tarugara et al., 2022). The higher variable importance of the land cover classes Forest and Woodland in 2020 is possibly related to higher canopy cover indicating lower *Lantana camara* invasion, as the species is sensitive to dense shadows cast by other plants (Bhatt et al., 2012).

In the subsetted model, there was a more evident spatial differentiation in habitat suitability between the western and eastern sections of the park, indicating that a focused set of predictors can yield more distinct and specific insights than models with numerous predictors (**Figure 6**). Additionally, the high consistency (low SDs in large areas) and high model performance metrics (mean AUC_ROC_ of 0.98 and AUC_PR_ of 0.78) validate the efficacy of the selected subset of predictors. The associated high SD of the Roads predictor highlights variability across model iterations, a constraint further discussed in section 4.2.

The temporal analysis of *Lantana camara* habitat suitability in Akagera National Park revealed notable spatial dynamics (**Table 4**). A positive, albeit not linear, trend in increasing habitat suitability is observed over the timeframe from 2015 to 2023, suggesting the influence of multiple interacting factors. Increases in central and northeastern areas suggest growing suitability for *Lantana camara.* In contrast, a decline in the southwest may reflect shifting environmental conditions or the impact of targeted management interventions, such as burning (**Figure 4**) (Ssali et al., 2024). Substantial increases in 2016 and 2023, particularly in the southern regions, indicate periods of favorable environmental conditions for *Lantana camara* growth. These temporal shifts appear closely linked to climatic predictors, particularly AET, PET, PR, and temperature-related predictors, which emerged as strong contributors in the predictive model. This is consistent with Seid and Bekele (2023) and Pagny et al. (2020), who also identified precipitation and temperature-related variables as key predictors in modeling the spatial distribution of *Lantana camara*.

### 4.2 Applicability and constraints of the model framework

While the model demonstrates high predictive performance, it is essential to assess the underlying constraints that may critically influence predictions. A key limitation of this study is the restricted availability of ground-truth data for *Lantana camara* in the study area, which was limited to 2023. This constraint affects the reliability of retrospective predictions and change detection analyses. While the SDM demonstrated high accuracy, the absence of ground-truth data for earlier years necessitates a cautious interpretation of these results. Additionally, the retrospective classifications assume that *Lantana camaras* ecological characteristics remained consistent throughout the study period. Annual ground-truth data would have been necessary to enhance the reliability of the findings and more accurately assess the habitat changes over time (Crego et al., 2023).

In addition to the temporal data limitations, spatial characteristics of the input data also introduce essential constraints. In the subset model run for 2023, the road network exhibited high SD values, indicating significant variation across different model scenarios (**Figure 6B**). The high variability may be attributed to spatial autocorrelation (SAC) of the predictors, a common issue in SDMs. SAC arises when nearby observations exhibit similar values, reducing the actual degrees of freedom and biasing the significance of explanatory predictors (Harisena et al., 2021; Paradinas et al., 2023). This effect is further amplified when using remotely sensed imagery, where resolution, extent, and ecological responses interact, enhancing SAC. Tobler's First Law of Geography, which states that "everything is related to everything else, but near things are more related than distant things" (Tobler, 1970), is particularly relevant here. The clustering of predicted suitable habitats around observation points highlights the influence of spatial proximity on occurrence likelihood (**Figures 1**, **3**). Given that ground-truth points were predominantly sampled near roads, this may have biased the model's responsiveness to the road network, inflating its relative importance and variability. To reduce SAC, the presence data were initially thinned to include only one randomly selected record per pixel, thereby minimizing pseudo-replication and mitigating spatial biases (Dormann et al., 2007; Crego et al., 2022). However, while this step improves model reliability, it cannot eliminate SAC, which remains a challenge when interpreting species-environment relationships (Koldasbayeva et al., 2024).

### 4.3 Future research and conservation implications

*Lantana camara* features various characteristics that contribute to its invasiveness, some of which could not be included in our model due to limited data availability. One important factor is its dispersal through animal vectors. Despite its high tannin content, which generally protects *Lantana camara* from herbivory, some wildlife species, such as browsing ungulates and primates, consume parts of the plant, facilitating its spread (Wronski et al., 2017). Additionally, the seeds of the IAS are primarily spread by a diverse array of bird species through migratory processes, enabling the species to expand its range and occupy new environments rapidly (Raghubanshi et al., 2005; Vardien et al., 2012; Wronski et al., 2017). Consequently, the distribution of *Lantana camara* is likely to correlate with areas of high wildlife density and species richness in savannah ecosystems (Wronski et al., 2017). Therefore, future studies could significantly benefit from incorporating animal dispersal mechanisms and animal density data.

The relationship between *Lantana camara* distribution and anthropogenic pressure merits deeper investigation through the inclusion of additional predictors. In the present study, the road network within the Akagera National Park served as the sole proxy for anthropogenic disturbance. It consistently emerged as a key driver of habitat suitability across all modelled years. Future studies should further refine this predictor by including a more advanced road layer with information such as road size and tourist accessibility. About anthropogenic pressures, Wronski et al. (2017) reported that intensified land use, driven by the return of refugees and the shift from pastoralism to subsistence farming in the Mutara rangelands bordering our study area, has contributed to the species spread. To enhance the predictive power and ecological relevance of future *Lantana camara* species distribution models, we therefore recommend incorporating a broader range of anthropogenic predictors, including land-use intensity, population density, and the proximity to settlements. 

Regarding future studies, further exploration of whether the annual time steps for the analysis were the most insightful intervals is advised, considering the growth rates and invasion dynamics of *Lantana camara*. While specific data for Rwanda or Eastern Africa are lacking, rapid growth rates (not numerically defined) of the IAS have been observed in other African savannah ecosystems as reported by several studies (Vardien et al., 2012; Goyal and Sharma, 2015; Ntalo et al., 2022). These findings suggest that shorter temporal intervals could provide additional insights into *Lantana camaras* response to regional management practices such as burning (Ssali et al., 2024). A seasonal analysis based on dry and wet seasons, as recently suggested by Mtyobila and Shoko (2024) for a South African ecosystem, could potentially serve as a suitable temporal unit for monitoring *Lantana camara* dispersion in Akagera National Park.

The observed sensitivity of habitat suitability to interannual climatic variation underscores the importance of incorporating future climate projections into IAS modeling. This would enable more robust predictions and support the long-term management of *Lantana camara* invasion risk under changing climate scenarios (Qin et al., 2016; Adhikari et al., 2024).

## 5 Conclusion

To our knowledge, this study represents the first effort to model habitat suitability and spatiotemporal invasion dynamics of *Lantana camara* in Akagera National Park, Rwanda, providing a foundation for both scientific advancement and practical conservation interventions in the area. The results highlight critical areas of vulnerability, particularly along the park’s road network and in low-lying central and northeastern regions, where ecological conditions and anthropogenic factors converge to support invasion. These insights are highly relevant for park management, offering spatially explicit guidance for monitoring, early intervention, and habitat restoration. The model identified elevation, soil nitrogen, and the proximity to the road network as key drivers of invasion, reflecting the importance of both environmental and anthropogenic pressures. Despite the high predictive performance of the species distribution model, it is essential to acknowledge its inherent limitations, particularly the limitation to field data exclusively from 2023 and the exclusion of biological dispersal mechanisms. Future research should aim to incorporate more detailed soil nutrient data, assess seasonal dynamics, and investigate the role of animal dispersal in shaping the spread of L*antana camara*. Incorporating predictive climate scenarios could further strengthen long-term management strategies by anticipating shifts in invasion risk under future environmental conditions. In conclusion, this study presents a comprehensive framework for modeling *Lantana camara* habitat suitability and spatiotemporal distribution patterns. It offers valuable insights for targeted conservation strategies and ecosystem management in Akagera National Park while also contributing to a broader understanding of *Lantana camara* invasion dynamics in East African ecosystems.

## Data Availability

The raw data supporting the conclusions of this article will be made available by the authors, without undue reservation.
